# Age‐Related and Seasonal Variation in Malaria and Other Causes of Fever and Their Association With Clinical Outcomes in Southern Angola: A Hospital‐Based Study

**DOI:** 10.1111/tmi.70166

**Published:** 2026-05-16

**Authors:** Helga E. M. Gonçalves, Maria Lina Antunes, Jorge Seixas, Marcelo U. Ferreira

**Affiliations:** ^1^ Global Health and Tropical Medicine (GHTM), Associate Laboratory in Translation and Innovation Towards Global Health (LA‐REAL) Institute of Hygiene and Tropical Medicine, Nova University of Lisbon Lisbon Portugal; ^2^ Faculty of Medicine Agostinho Neto University Luanda Angola; ^3^ Central Hospital Dr. António Agostinho Neto Lubango Huíla Angola; ^4^ Department of Parasitology Institute of Biomedical Sciences, University of São Paulo São Paulo São Paulo Brazil

**Keywords:** Angola, clinical outcomes, febrile illness, malaria, nonmalarial fever, seasonal variation

## Abstract

**Background:**

Acute febrile illnesses in sub‐Saharan Africa are often attributed to malaria, yet many patients test negative for malaria parasites. The aetiology of nonmalarial fevers remains understudied. Here, we examine likely causes of febrile illnesses and their association with poor clinical outcomes in Angola.

**Methods:**

In September–October 2023 and May–June 2024 we enrolled 849 febrile patients with ages between 2 months and 94 years in two tertiary hospitals of Lubango city that serve 7 million people in southern Angola. We used clinical assessments and locally available laboratory diagnostics to assign likely causes of fever and applied logistic regression analysis to identify factors independently associated with an increased risk of hospital admission and death.

**Results:**

Overall, 67.6% of the patients tested negative for malaria by microscopy, with upper (13.0%) and lower (19.5%) respiratory infections and gastrointestinal infections (16.7%) being leading causes of nonmalarial fever. The frequencies of gastrointestinal, upper respiratory, and genitourinary infections varied markedly with age, and malaria predominated at the beginning of the dry season. Malaria and lower respiratory infections were associated with increased risk of hospitalization (odds ratio [OR] = 3.20 and 5.17, *p* < 0.0001), while malaria and central nervous system infections were linked to significantly higher mortality (OR = 1.90 and 6.30, *p* < 0.0001). Empirical antibiotic use was widespread, even without clinical or laboratory evidence of bacterial infection.

**Conclusion:**

Most fevers in southern Angola are of nonmalarial origin, underscoring the need for better diagnostic strategies to guide specific treatment and reduce unnecessary antibiotic use.

## 1 Background

Nowadays, microscopy and point‐of‐care rapid diagnostic tests for malaria are commonplace in health facilities across sub‐Saharan Africa [1]. Malaria can now be confirmed and treated appropriately [2], but clinicians face the challenge of managing an increasing proportion of febrile patients who test negative for malaria parasites.

Understanding how the aetiology of febrile illnesses in sub‐Saharan Africa varies with age, season and geographic setting is essential for improving clinical management [3]. However, in resource‐constrained settings causes of nonmalarial fevers are often difficult to distinguish based solely on clinical presentation [4] and patients frequently receive antimalarials (e.g., [5]) or antibiotics empirically (e.g., [6]), regardless of negative laboratory test results for malaria and bacterial infection [3].

Most febrile under‐five children who test negative for malaria in outpatient facilities across the continent [4, 7], and of those who are seen at home [8], have acute respiratory tract infections, predominantly of viral origin. Relatively few have radiologically confirmed pneumonia [9]. Additionally, 10%–25% of febrile children have gastroenteritis [8], while the remaining cases are mostly due to urinary tract infections (1%–6%) and other localized infections, such as central nervous system and skin/soft‐tissue infections (< 5%) [9]. Nonmalarial fevers in older children and adults are often associated with HIV infection, leptospirosis, rickettsiosis, scrub typhus and brucellosis, as well as acute lower respiratory tract infections [9].

The causes of nonmalarial febrile illnesses are understudied in Angola, with only four case series identified through a systematic review of 1065 articles focusing on Africa published between 1985 and 2015 [3]. Likewise, a systematic review and meta‐analysis of 133 studies on fever aetiology across Africa, published before December 2018, included none from Angola [7]. To further assess the literature, we conducted a PubMed search using the same terms as Wainaina et al. [7], substituting ‘Africa’ with ‘Angola’ and found a single article published between 2006 and 2025 that addressed the aetiology of febrile illnesses in the country. This study reported serological evidence of *Rickettsia* infection in 3 (3.5%) of 87 febrile Angolan patients studied between February 2016 and March 2017 who tested negative for malaria and yellow fever [10].

Here, we examine the association between clinical syndromes and poor outcomes in febrile patients from southern Angola, with the aim of informing the management of nonmalarial fevers in this and other similar settings in Africa.

## 2 Methods

### 2.1 Study Design

This is a hospital‐based observational study.

### 2.2 Study Setting

Lubango city, with 1,400,000 inhabitants, is the capital of Huíla Province (population, 3.1 million). We enrolled febrile patients presenting at the emergency room of two tertiary hospitals of Lubango: the Central Hospital Dr. Agostinho Neto (418 beds and a daily average of 180 patients attending the emergency room) and the Children's Hospital Pioneiro Zeca (180 beds and a daily average of 130 patients aged < 16 years attending the emergency room). Their catchment area encompasses the provinces of Huíla, Cuando Cubango, Cunene, and Namibe, with a total of approximately seven million inhabitants.

Malaria is mesoendemic and seasonal in Huíla [11], with an average prevalence of infection of 11.2% among under‐five children in 2023–2024 [12]. The prevalence of serologically confirmed HIV infection among men and women aged 15–49 years was estimated at 0.6% in 2023–2024 [12].

### 2.3 Inclusion and Exclusion Criteria

Between 7 September and 27 October 2023 (beginning of the rainy season) and between 12 May and 20 June 2024 (beginning of the dry season), participants were recruited at the emergency room if they met the following criteria: (a) age ≥ 2 months, (b) axillary temperature ≥ 37.5°C measured at the emergency room or reported fever within the past 48 h, (c) willingness and ability to provide demographic and clinical information at the time of enrolment and 28 days later, and (d) willingness and ability to provide informed consent. A recent visit to a health care facility did not constitute an exclusion criterion. However, because we focus on community‐acquired infections, we excluded patients with a hospital admission (duration > 24 h) within the past 14 days due to the risk of nosocomial infection.

### 2.4 Sample Size Estimation

We used the Scalex SP calculator [13] to estimate that at least 401 participants were needed per hospital, for an absolute precision of ±2% to estimate the frequency of clinical entities that affected ≥ 3% of the patients, considering a loss of day‐28 follow‐up information for 30% of the study participants.

### 2.5 Data Collection

At enrollment, participants were interviewed and examined by a study physician (HEMG), who used a standardized case report form to record information on (a) patient's sex and age; (b) pregnancy status for women aged 15–49 years; (c) fever duration; (d) current or recent diarrhoea, vomiting, coughing, and any other symptoms and signs, including those suggestive of severe malaria; and (e) previous treatment, at home or another health facility. Weight and height were measured at enrollment to calculate weight‐for‐age and height‐for‐age *z* scores [14] and body mass index‐for‐age *z* scores [15] of children aged < 5 and children and adolescents aged 5–19 years, respectively. Participants ≥ 20 years of age had their nutritional status assessed on the basis of their body mass index.

Laboratory support included automated blood cell counts; basic clinical biochemistry; rapid diagnostic tests for malaria, typhoid fever, dengue, and COVID‐19; and serology for typhoid fever (Widal test) and viral hepatitis. Chest and abdominal x‐rays, ultrasonography, and computed tomography were available at the Central Hospital only. All participants were screened for malaria parasites by thick‐smear microscopy; at least 100 fields were examined under 1000× magnification before a slide was declared negative.

### 2.6 Syndromic Categories Defined at Enrollment

Malaria was considered a potential cause of fever in patients with a positive microscopy, regardless of the parasitemia or evidence of co‐infection with other pathogens. Based on clinical and laboratory information available at admission, the study physician assigned patients to one or more of the following syndromic categories (modified from [16]): (a) upper respiratory tract infection (nasal congestion, rhinorrhea, or sore throat or swollen tonsils with or without white patches or pus, and/or cough/sneezing but no clinical signs of pneumonia); (b) lower respiratory tract infection (productive cough > 3 days of duration, or discoloured sputum, or physical examination and/or chest x‐ray consistent with pneumonia, or respiratory rate > 20/min for adults and above age‐stratified cut‐off values for paediatric patients); (c) gastrointestinal infection including hepatitis (stool frequency > 3 per day, or loose/liquid/blood‐stained stools on at least 1 day prior to enrollment, or abnormalities on abdominal examination consistent with gastrointestinal infection and its complications, or jaundice in the absence of malaria and/or elevated alanine transaminase or aspartate transaminase levels); (d) genitourinary infection (increased urinating frequency, or dysuria, or flank pain, or lower abdomen/pelvic tenderness or genital lesion on physical examination); (e) central nervous system infection (any sign or symptom of central nervous system infection, including neck stiffness and altered consciousness); (f) skin/joint/mucosal infection (e.g., cutaneous lesion, or joint inflammation and/or effusion consistent with infectious arthritis, or mucosal infection, or purulent ear discharge, or dental infection/abscess); (h) fever with another focus or no focus identified (no sign of malaria or respiratory, gastrointestinal, genitourinary, central nervous system, or skin/joint/mucosal infection).

### 2.7 Patient Follow‐Up

Study participants returned to the hospital for reassessment, or were interviewed over a phone call, approximately 28 days after enrollment. At the 28‐day follow‐up visit, the study physician defined the clinical outcome as: (a) complete recovery, (b) improvement but incomplete recovery, (c) worse than on day 0, (d), death, or (e) loss to follow‐up (modified from [17]). Information regarding hospital admission or death from patients who were transferred to another health facility or could not be reached was obtained from another household member. The final diagnosis assigned to patients by the attending clinician at discharge, which often included a likely etiologic diagnosis (e.g., typhoid fever instead of gastrointestinal infection), was also recorded.

### 2.8 Statistical Analysis

Data were analysed using Stata 18.0 software, with statistical significance defined at the 5% level. Standard χ^2^ tests were used to compare proportions, and logistic regression analysis was employed to identify factors associated with the risk of hospital admission and death. Covariates associated with the outcome at a significance level of < 20% in unadjusted analysis were retained in the final multivariable logistic regression models. Separate logistic regression models were fitted for each syndromic category. Odds ratios (ORs), along with their 95% confidence intervals (CIs), were used to quantify the magnitude of associations between exposures and outcomes, while controlling for potential confounders. We used the Benjamini‐Hochberg procedure [18] to control for the false discovery rate (*q*) when *m* comparisons were made in the same sample set. We set *q* at 0.10, meaning that we accept up to 10% of the associations with significant results being false positives. This article adhered to the Strengthening the Reporting of Observational Studies in Epidemiology (STROBE) reporting guidelines.

## 3 Results

### 3.1 Characteristics of the Study Population

We enrolled 849 patients, 45.3% females, with ages between 2 months and 94 years (average, 19.8 years). Participant's characteristics and syndromic categories defined at the emergency room visit are shown in Table 1. Only one‐third of the participants tested positive for malaria. Malaria‐positive patients often reported a previous primary care facility visit (36.0%; 99 of 275) and pre‐referral use of antimalarials (22.2%; 61 of 275); 41 (14.9%) of them had other infectious diagnoses, for example, lower respiratory tract infections (16 cases; 5.8%) or gastrointestinal/hepatic infections (11 cases; 4.0%).

**TABLE 1:** Demographic characteristics, clinical presentation, and outcomes of study participants, Lubango city, Southern Angola, 2023–2024.

Characteristic	Frequency (%)
Age (years)	
< 5	259 (30.5%)
5 to 14	165 (19.4%)
15 to 29	201 (23.7%)
30 to 44	123 (14.5%)
≥ 45	101 (11.9%)
Sex	
Female	385 (45.3%)
Male	464 (54.6%)
Hospital	
Children's Hospital Pioneiro Zeca	411 (48.4%)
Central Hospital Dr. Agostinho Neto	438 (51.6%)
Month of enrollment	
September 2023	260 (30.6%)
October 2023	241 (28.4%)
May 2024	285 (33.6%)
June 2024	63 (7.4%)
Pre‐hospitalar care since symptom onset	
Previous visit to primary health care facility	286 (33.7%)
Use of antipyretics	537 (63.2%)
Use of antimalarials	140 (16.5%)
Use of antibiotics	177 (20.8%)
Fever	
Duration of reported fever (median [range], days)	4 (< 1 to 60) days
Fever (axillary temperature ≥ 37.5°C) at presentation	790 (93.0%)
HIV infection status	
Self‐reported HIV‐positive status	17 (2.0%)
Positive rapid diagnostic test (RDT) for HIV at admission	32 (3.8%)
Self‐reported or positive RDT at admission	37 (4.4%)
Nutritional anthropometry	
Children 0–4.9 years (weight data for *n* = 254 and height data for *n* = 247)	
Wasting (weight‐for‐age *z* score < −2.0)	105 (41.3%)
Stunting (height‐for‐age *z*–score < −2.0)	131 (53.0%)
Children and adolescents 5–19.9 years (weight and height data for *n* = 229)	
Underweight (BMI‐for‐age *z* score < −2)	52 (22.7%)
Adequate weight (BMI‐for‐age *z* score from −2.0 to 0.9)	171 (74.7%)
Overweight (BMI‐for‐age *z* score from 1.0 to 1.9)	4 (1.8%)
Obesity (BMI‐for‐age *z* score ≥ 2.0)	2 (0.9%)
Adults ≥ 20 years (weight and height data for *n* = 338)	
Underweight (BMI < 18.5)	82 (24.3%)
Adequate weight (BMI from 18.5 to 24.9)	199 (58.9%)
Overweight (BMI from 25.0 to 29.9)	46 (13.6%)
Obesity (BMI ≥ 30.0)	11 (3.2%)
Cause of fever identified at enrollment^a^	
Upper respiratory tract infection	110 (13.0%)
Lower respiratory tract infection	166 (19.5%)
Gastrointestinal or hepatic infection	142 (16.7%)
Genitourinary infection	43 (5.1%)
Central nervous system infection	22 (2.5%)
Skin/joint/mucosal infection	38 (4.5%)
Fever with another focus or no focus identified	103 (12.1%)
Positive microscopy for malaria	275 (32.4%)
28‐day follow‐up	
Complete recovery	558 (68.2%)
Partial recovery	157 (18.5%)
Death during hospital stay	103 (12.3%)
Transferred to another hospital	31 (3.6%)
Final outcome	
Discharge without admission	397 (46.8%)
Admission to the study hospital or another hospital	452 (53.2%)
Death at the study hospital or another hospital	108 (12.7%)

^a^ One patient may be classified into two or more clinical syndromes.

**FIGURE 1:**
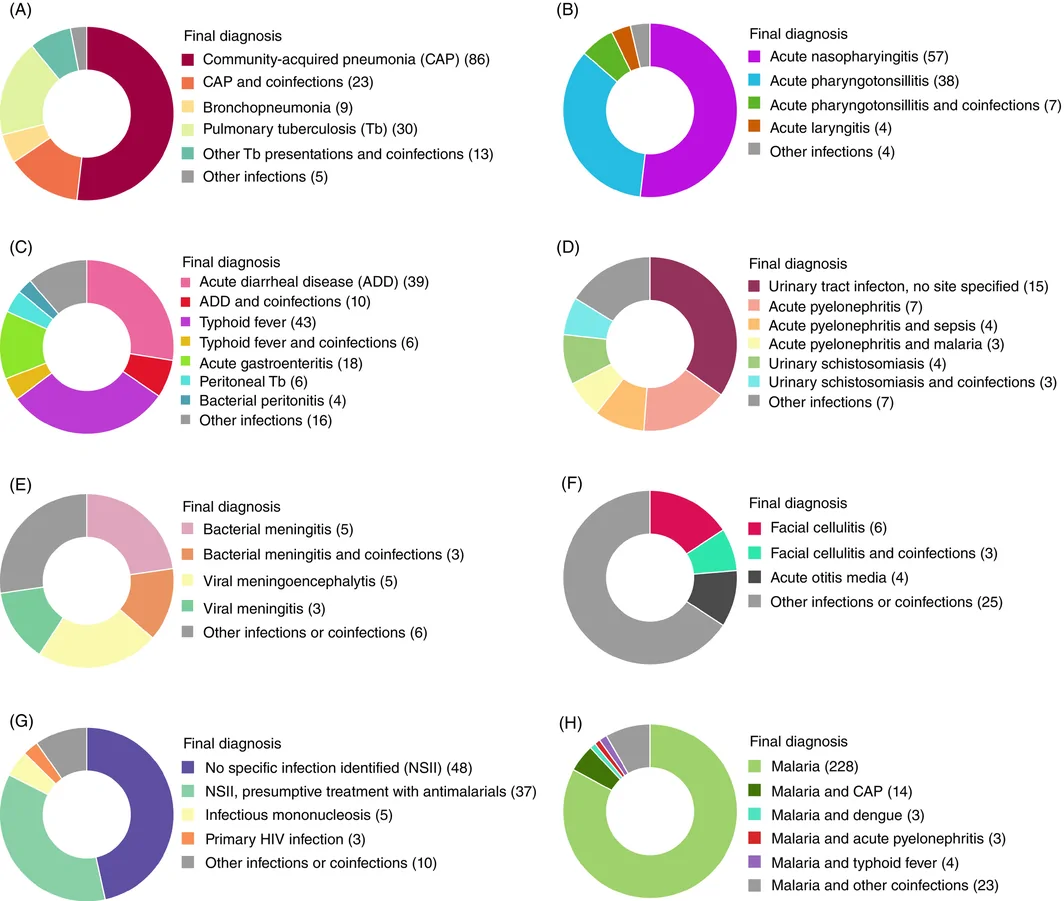
Correspondence between syndromic categories assigned by the study physician at enrollment and final diagnoses assigned by the attending physician at discharge to 849 febrile patients seen at the Central Hospital Dr. Agostinho Neto and the Children's Hospital Pioneiro Zeca, Lubango, Angola, 2023–2024. Each panel shows data for a specific clinical syndrome: (A) Lower respiratory tract infection (*n* = 166 patients); (B) Upper respiratory tract infection (*n* = 110); (C) Gastrointestinal or hepatic infection (*n* = 142); (D) Genitourinary infection (*n* = 43); (E) Central nervous system infection (*n* = 22); (F) Skin/joint/mucosal infection (*n* = 38); (G) Fever with another focus or no focus identified (*n* = 103); and (H) Positive microscopy for malaria (*n* = 275). The same patient may have been assigned to more than one clinical syndrome. Table S1 shows all final diagnoses according to clinical syndrome.

Final diagnoses are listed in Table S1, while Figure 1 shows the correspondence between clinical syndromes at enrollment and final diagnoses at discharge. The most frequent diagnoses were malaria, with or without co‐infections (275 cases; 32.4%), community‐acquired pneumonia (107 cases; 12.6%), acute nasopharyngitis (57 cases; 6.7%), typhoid fever (49 cases [5.8%], nine of which with intestinal perforation), acute diarrheal disease (49 cases; 5.8%), acute pharyngotonsillitis (45 cases; 5.3%), and pulmonary tuberculosis (37 cases; 4.3%). The most common co‐infections were community‐acquired pneumonia and malaria (14 cases), acute diarrheal disease and HIV infection (6 cases), typhoid fever and malaria (4 cases), dengue and malaria (3 cases), and acute pyelonephritis and malaria (3 cases; Table S1). Only one pregnant woman participated in the study; she was 18 years‐old and received a final diagnosis of fever of unknown origin. Presumed etiologic diagnoses were made for 14 (13.6%) out of 103 cases initially classified as ‘fever with another focus or no focus identified’, while no specific infection was identified in 89 of 849 (10.5%) patients enrolled. Importantly, 37 (41.1%) of them were presumptively treated with antimalarials, despite the negative microscopy.

All 351 patients with a final diagnosis of bacterial infection received antibiotics, but 78 (15.7%) of 498 patients with fever of nonbacterial or unknown origin received pre‐referral antibiotics—most commonly amoxicillin/clavulanate (*n* = 35), metronidazole (*n* = 6), amoxicillin (*n* = 5), azithromycin (*n* = 4), and ciprofloxacin (*n* = 4). Pre‐referral antibiotics were often prescribed to patients with nonbacterial central nervous system infection (3 of 13, 23.1%), no focus identified (20 of 100; 20.0%), and malaria (44 of 244; 18.0%).

In addition, 353 (70.9%) of 498 patients with no evidence of bacterial (co)infection received antibiotics at the hospital—most commonly clindamycin (*n* = 101), amoxicillin (*n* = 58), azithromycin (*n* = 34), ciprofloxacin (*n* = 33), doxycycline (*n* = 26), ceftriaxone (*n* = 16), and amoxicillin/clavulanate (*n* = 13). Despite the lack of evidence of bacterial infection, antibiotics were often administered to patients with gastrointestinal infection (61 of 66, 92.4%), central nervous system infection (11 of 13, 84.6%), positive microscopy for malaria (172 of 244; 70.5%), upper respiratory tract infection (43 of 66; 68.2%), and those without a focus identified (62 of 100; 62.0%).

### 3.2 Age‐Related and Seasonal Variation in the Frequency of Syndromic Categories

The frequency of microscopy‐confirmed malaria, the most common likely cause of fever across all age groups, ranged from 26.2% (68 of 259) in under‐five children to 39.3% (79 of 201) in adolescents and young adults (Table 2). Upper respiratory tract infections were much more frequent among under‐five children (17.8%; 46 of 259) than in adults aged ≥ 45 years (4.9%; 5 of 101). Likewise, gastrointestinal or hepatic infections were identified in one‐quarter of the under‐five children, but their frequency decreased in the older age groups. Conversely, genitourinary infections were six‐fold more frequent in adults aged ≥ 45 years (11.9%; 12 of 101) than in under‐five children (1.9%; 5 of 259).

**TABLE 2:** Cause of fever according to patient's age, Lubango city, Southern Angola, 2023–2024.

Cause of fever identified at enrollment	Frequency (%) by age group (years)						*p* (χ^2^ test)
	< 5	5 to 14	15 to 29	30 to 44	≥ 45.0	Total	
Upper respiratory tract infection	**46 (17.8%)**	23 (13.9%)	22 (10.9%)	14 (11.4%)	5 (4.9%)	110 (12.7%)	0.016
Lower respiratory tract infection	45 (17.4%)	**39 (23.6%)**	**34 (16.9%)**	**24 (19.5%)**	**24 (23.8%)**	**166 (23.8%)**	0.336
Gastrointestinal or hepatic infection	**66 (25.5%)**	**24 (14.6%)**	24 (11.9%)	15 (12.2%)	**13 (12.9%)**	**142 (16.7%)**	< 0.0001
Genitourinary infection	5 (1.9%)	5 (3.0%)	10 (5.0%)	11 (8.9%)	12 (11.9%)	43 (5.1%)	< 0.0001
Central nervous system infection	6 (2.3%)	5 (3.0%)	6 (3.0%)	2 (1.6%)	3 (3.0%)	22 (2.6%)	0.933
Skin/joint/mucosal infection	17 (6.6%)	8 (4.8%)	5 (2.5%)	5 (4.1%)	3 (3.0%)	38 (4.5%)	0.274
Fever with another focus or no focus identified	24 (9.3%)	21 (12.7%)	**28 (13.9%)**	**18 (14.6%)**	12 (11.9%)	103 (11.9%)	0.495
Positive microscopy for malaria	**68 (26.2%)**	**48 (29.1%)**	**79 (39.3%)**	**43 (35.0%)**	**37 (36.6%)**	**275 (32.4%)**	0.028
Total	259	165	201	123	101	849	

*Note:* Frequencies of the three most common diagnoses in each age group are indicate bold values.

We found significant seasonal variation in the relative frequency of specific clinical syndromes (Table 3). Lower respiratory tract infections and gastrointestinal or hepatic infections were common likely causes of fever at the beginning of the rainy season, in September–October 2023, while malaria was diagnosed in only 82 (16.4%) of the 501 participants enrolled during the rainy season of 2023. By contrast, the majority (55.5%; 193 of 348) of patients enrolled during the subsequent dry season (May–June 2024) harboured malaria parasites. The relative frequency of other infections remained relatively unchanged over the study period.

**TABLE 3:** Cause of fever according to patient's month of enrollment, Lubango city, Southern Angola, 2023–2024.

Cause of fever identified at enrollment	Frequency (%) by month of enrollment					*p* (χ^2^ test)
	September 2023	October 2023	May 2024	June 2024	Total	
Upper respiratory tract infection	36 (13.8%)	**45 (18.7%)**	26 (9.1%)	3 (4.8%)	110 (13.0%)	0.002
Lower respiratory tract infection	**67 (25.8%)**	**54 (32.5%)**	**37 (22.3%)**	**8 (12.7%)**	**166 (19.6%)**	0.001
Gastrointestinal or hepatic infection	**45 (17.3%)**	**52 (21.6%)**	**34 (11.9%)**	**11 (17.5%)**	**142 (16.7%)**	0.031
Genitourinary infection	24 (9.2%)	7 (2.9%)	9 (3.2%)	3 (4.8%)	43 (5.1%)	0.003
Central nervous system infection	7 (2.7%)	7 (2.9%)	7 (2.5%)	1 (1.6%)	22 (2.6%)	0.945
Skin/joint/mucosal infection	13 (5.0%)	19 (7.9%)	6 (2.1%)	0 (0.0%)	38 (4.5%)	0.004
Fever with another focus or no focus identified	**41 (15.8%)**	28 (11.6%)	27 (9.5%)	7 (11.1%)	103 (12.1%)	0.155
Positive microscopy for malaria	38 (14.6%)	44 (18.3%)	**158 (55.4%)**	**35 (55.6%)**	**275 (32.4%)**	< 0.0001
Total	260	241	285	63	849	

*Note:* Frequencies of the three most common diagnoses in each month are indicated in bold values.

### 3.3 Syndromic Categories in Relation to Clinical Outcomes

Clinical outcomes at day 28 varied according to the syndromic category defined at the emergency room (Table S2). The majority of patients with upper respiratory tract infections (98.2%; 107 of 109), microscopy‐confirmed malaria (81.3%; 222 of 273), genitourinary infections (79.1%; 34 of 43), skin/joint/mucosal infections (78.9%; 30 of 38), and gastrointestinal or hepatic infections (68.1%; 94 of 138) had a complete recovery by day 28, while 61 of 102 (59.8%) patients with fever without a source or another source reported only a partial recovery. Ten of 22 (45.4%) patients with central nervous system infection had died by Day 28.

Overall, 452 (53.2%) febrile patients seen at the emergency room were admitted to the study hospitals or other health facilities for further investigation and treatment; 108 (12.7%) participants died during the hospital stay (Table 1). Patients enrolled during the dry season (68.1%; 237 of 348) and carriers of HIV infection (75.7%; 28 of 37) had a comparatively higher hospitalization rate. Likewise, patients enrolled after September 2023 (16.3%; 96 of 589), children aged < 15 years (17.9%; 76 of 424) and admitted to the Children's Hospital (18.5%; 76 of 411), and carriers of HIV infection (37.8%; 14 of 37) had a comparatively higher mortality rate during the hospital stay. Odds ratios and 95% CIs are shown in Table 4.

**TABLE 4:** Demographic and morbidity variables associated with the risk of hospital admission and death, Lubango city, Southern Angola, 2023–2024.

Variable	No.	No. (%) admitted	No. (%) deaths	Unadjusted logistic regression models			
				Admission to hospital		Death	
				OR (95% CI)	*p*	OR (95% CI)	*p*
Age (years)							
< 5	259	125 (48.3%)	42 (16.2%)	1 (reference)		1 (reference)	
5 to 14	165	93 (56.4%)	34 (20.6%)	1.38 (0.93–2.05)	0.104	1.34 (0.81–2.21)	0.252
15 to 29	201	113 (56.2%)	15 (13.9%)	1.38 (0.95–1.99)	0.091	0.42 (0.22–0.77)	0.006
30 to 44	123	64 (52.0%)	9 (7.3%)	1.16 (0.76–1.79)	0.491	0.41 (0.19–0.87)	0.020
≥ 45	101	57 (56.4%)	8 (7.9%)	1.39 (0.87–2.21)	0.164	0.44 (0.20–0.98)	0.045
Sex							
Female	385	194 (50.4%)	51 (13.2%)	1 (reference)		1 (reference)	
Male	464	258 (55.6%)	57 (12.3%)	1.23 (0.94–1.62)	0.130	0.92 (0.61–1.37)	0.675
Hospital							
Children's Hospital Pioneiro Zeca	411	206 (50.1%)	76 (18.5%)	0.78 (0.60–1.03)	0.078	2.88 (1.86–4.46)	< 0.0001
Central Hospital Dr. Agostinho Neto	438	246 (56.2%)	32 (7.3%)	1 (reference)		1 (reference)	
Month of enrollment							
September 2023	260	110 (42.3%)	12 (4.6%)	1 (reference)		1 (reference)	
October 2023	241	105 (43.6%)	29 (12.0%)	1.05 (0.74–1.50)	0.776	2.83 (1.41–5.68)	0.003
May 2024	285	184 (64.6%)	52 (18.2%)	2.48 (1.75–3.51)	< 0.0001	4.61 (2.40–8.86)	< 0.0001
June 2024	63	53 (84.1%)	15 (23.8%)	7.22 (3.52–14.83)	< 0.0001	6.46 (2.84–14.66)	< 0.0001
HIV infection status							
Positive	37	28 (75.7%)	14 (37.8%)	2.84 (1.32–6.11)	0.007	4.65 (2.31–9.34)	< 0.0001
Negative or unknown	812	424 (52.2%)	94 (11.6%)	1 (reference)		1 (reference)	
Total	849	452 (53.2%)	108 (12.7%)				

*Note:* Odds ratios (OR) are shown along with their 95% confidence intervals (CIs) to quantify the magnitude of associations between exposures and outcomes (hospitalization or death), before (model 0) and after (model 1) adjusting for potential confounders.

We used multivariable logistic regression analysis to identify the clinical syndromes that were significantly associated with higher hospital admission (Table 5) and case fatality rates (Table 6). Separate models were run for each clinical entity and outcome. All 22 patients with a diagnosis of central nervous system infection were hospitalized. In addition, microscopy‐confirmed malaria and lower respiratory tract infection were also associated with a significantly increased risk of hospitalization (OR = 3.20 and 5.17, respectively, *p* < 0.0001; Table 5). Importantly, these associations remained statistically significant after adjusting for hospital, month of enrollment, age and HIV infection status, which were identified as potential confounders by unadjusted analysis. Microscopy‐confirmed malaria and central nervous system infection were associated with a higher case fatality rate, after adjusting for potential confounders, than other likely causes of fever (OR = 1.90 and 6.30, respectively, *p* < 0.0001; Table 6).

**TABLE 5:** Causes of fever and risk of hospital admission, Lubango city, Southern Angola, 2023–2024.

Cause of fever identified at enrollment	No.	No. (%) admitted	Logistic regression model			
			Model 0 (unadjusted)		Model 1 (adjusted)	
			OR (95% CI)	*p*	OR (95% CI)	*p*
Upper respiratory tract infection	110	3 (2.7%)	0.02 (0.01–0.06)	**< 0.0001**	0.02 (0.01–0.06)	**< 0.0001**
Lower respiratory tract infection	166	128 (77.1%)	3.73 (2.52–5.52)	**< 0.0001**	5.17 (3.40–7.85)	**< 0.0001**
Gastrointestinal or hepatic infection	142	82 (57.7%)	1.24 (0.86–1.79)	0.239	1.46 (0.99–2.16)	0.054
Genitourinary infection	43	12 (27.9%)	0.32 (0.16–0.64)	**0.001**	0.34 (0.16–0.72)	**0.005**
Central nervous system infection	22	22 (100.0%)	Not calculated		Not calculated	
Skin/joint/mucosal infection	38	16 (42.1%)	0.63 (0.32–1.21)	0.163	0.61 (0.30–1.24)	0.172
Fever with another focus or no focus identified	103	24 (23.3%)	0.23 (0.14–0.36)	**< 0.0001**	0.21 (0.13–0.36)	**< 0.0001**
Positive microscopy for malaria	275	207 (75.3%)	4.09 (2.97–5.63)	**< 0.0001**	3.20 (2.24–4.57)	**< 0.0001**
Total	849	452 (53.2%)				

*Note:* Odds ratios (OR) are shown along with their 95% confidence intervals (CIs) to quantify the magnitude of associations between exposures and the outcome (hospitalization), before (model 0) and after (model 1) adjusting for hospital, month of enrollment, age group, and HIV infection status. *p* values that remained significant after controlling for a false discovery rate (*q*) set at 0.10 are indicated in boldface (number of comparisons *m* = 7).

**TABLE 6:** Causes of fever and risk of death during hospital stay, Lubango city, Southern Angola, 2023–2024.

Cause of fever identified at enrollment	No.	No. (%) deaths	Logistic regression model			
			Model 0 (unadjusted)		Model 1 (adjusted)	
			OR (95% CI)	*p*	OR (95% CI)	*p*
Upper respiratory tract infection	110	0 (0.0%)	Not calculated		Not calculated	
Lower respiratory tract infection	166	27 (16.3%)	1.44 (0.90–2.32)	0.128	1.58 (0.93–2.66)	0.088
Gastrointestinal or hepatic infection	142	16 (11.3%)	0.85 (0.48–1.49)	0.569	0.73 (0.40–1.34)	0.314
Genitourinary infection	43	3 (7.0%)	0.50 (0.15–1.65)	0.255	0.71 (0.20–2.55)	0.602
Central nervous system infection	22	10 (45.5%)	6.20 (2.61–14.73)	**< 0.0001**	6.30 (2.44–16.28)	**< 0.0001**
Skin/joint/mucosal infection	38	3 (7.9%)	0.58 (0.17–1.91)	0.367	0.44 (0.12–1.65)	0.224
Fever with another focus or no focus identified	103	12 (11.6%)	0.89 (0.47–1.69)	0.728	1.01 (0.51–2.01)	0.969
Positive microscopy for malaria	275	49 (17.8%)	1.89 (1.26–2.85)	**0.002**	1.90 (1.18–3.04)	**0.008**
Total	849	108 (12.7%)				

*Note:* Odds ratios (OR) are shown along with their 95% confidence intervals (CIs) to quantify the magnitude of associations between exposures and the outcome (death), before (model 0) and after (model 1) adjusting for hospital, month of enrollment, age group, and HIV infection status. *p* values that remained significant after controlling for a false discovery rate (*q*) set at 0.10 are indicated in boldface (number of comparisons *m* = 7).

## 4 Discussion

Febrile illnesses are a frequent reason to visit health facilities across sub‐Saharan Africa, but clinical definitions vary widely and etiologic diagnosis is typically limited to a few pathogens for which point‐of‐care diagnostic methods are available [7]. Here, we analyse 849 emergency room visits for fever in tertiary hospitals in southern Angola, with a hospitalization rate of 53.2% and a case fatality rate of 12.7%. Overall, more than two‐thirds of the patients tested negative for malaria parasites, with little variation with age, consistent with the estimates of malaria‐attributable and nonmalarial fever in sub‐Saharan African under‐five children derived from spatiotemporal modelling of household survey data from 2006 to 2016 [19, 20].

The widespread availability of point‐of‐care diagnosis has increased the proportion of correctly treated malaria patients, but a high proportion of nonmalarial fevers are treated with antibiotics (e.g., [6]). Accordingly, 70.9% of our patients with fever of nonbacterial or unknown origin received empirical antibiotics during their hospital stay, an overuse pattern that may drive antimicrobial resistance [3, 21]. Most nonmalarial fevers were associated with lower respiratory tract infections, gastrointestinal infections, or upper respiratory tract infections. Typhoid fever, a common yet relatively neglected disease in sub‐Saharan Africa [22], was particularly frequent (5.8% of all febrile illnesses) and severe (9 of 49 patients had intestinal perforation). Of note, *Salmonella Typhi* resistance to ciprofloxacin, the antibiotic most commonly used to treat typhoid fever in Lubango hospitals, appears to have already emerged in Angola [23]. Fever without a source was diagnosed by attending clinicians in only 10.6% of patients, despite the limited availability of diagnostic tools. Similar to previous studies [4, 7, 21, 24], we observed a clear age‐related variation in the frequencies of specific clinical syndromes, such as gastrointestinal, upper respiratory, and genitourinary infections.

The relative frequency of malaria and clinical syndromes associated with nonmalarial fever also varied with season. Because Huíla province experiences malaria epidemics toward the end of the rainy season, from January to April [11], we hypothesize that patients enrolled at the beginning of the dry season (May–June 2024) might still be affected by the increased transmission that occurred a few months earlier. However, further research is needed to investigate the month‐by‐month variation in the aetiology of local fevers.

Finding a case fatality rate for microscopy‐proven malaria as high as 17.8% was somewhat surprising, given the access to artemisinin combination therapies (ACT) [12, 25] and pre‐referral antimalarials (Table 1). We suggest that primary care facilities are referring their more severe malaria cases to tertiary hospitals [26]. Moreover, other locally prevalent pathogens may aggravate malaria (e.g., [27, 28]) and 14.9% of our microscopy‐positive patients had co‐infections. Finally, we speculate that some patients may have failed to respond to ACT administered before or during hospitalization, as *Plasmodium falciparum* partial resistance to ACT appears to be emerging in Angola. There are repeated reports of reduced ACT efficacy in Angola [29, 30, 31] and parasites carrying two or more copies of the *P. falciparum multidrug resistance 1* (*pfmdr1*) gene appear to have been selected by artemether‐lumefantrine, the most commonly used ACT [32]. Accordingly, parasites with multiple copies of the *pfmdr1* gene were recently observed in 10% of the patients attending the Central Hospital of Lubango [33].

### 4.1 Study Strengths and Limitations

This study has key strengths, such as the large sample size and the use of standardized criteria to assign patients to syndromic categories. However, we acknowledge five main limitations. First, our sample may have been affected by substantial referral bias toward more severe cases, which greatly limits the generalizability of our findings to patients attending primary care facilities. Second, our results may not be applicable to other regions of Angola, where the relative contributions of malaria [11] and other endemic infections to acute febrile illnesses remain largely unknown. Third, we enrolled participants within two discrete time windows, rather than year‐round, which may reduce the strength of conclusions regarding seasonal variation. Forth, as in other resource‐constrained settings [4, 7, 21], we were unable to systematically investigate the aetiology of all fevers using culture, sequential serology, and molecular tests. This is the case of typhoid fever, whose diagnosis relied primarily on the Widal test and rapid diagnostic tests for detection of specific IgG and IgM antibodies and antigen on blood samples, but rarely on blood or stool culture. As a result, discharge diagnoses based on clinical findings and limited laboratory testing must be interpreted with caution. Importantly, incomplete etiologic investigation may lead to an overestimation of malaria‐related morbidity in settings where most malarial infections are actually asymptomatic, while undetected pathogens coincident with a malarial infection and likely to cause fever are underreported [19, 20]. Fifth, 43 (5.0%) participants had fever for more than 2 weeks, and their condition could not be strictly defined as an ‘acute febrile illness’.

### 4.2 Study Implications

Our findings underscore the need for improved diagnostics to assign patients to well‐defined clinical syndromes at the emergency room, to guide targeted treatment of febrile illnesses and reduce unnecessary antibiotic use in healthcare settings of sub‐Saharan Africa.

## 5 Conclusion

We found that the majority of febrile patients seeking care in emergency rooms in southern Angola tested negative for malaria parasites, but the overall contribution of malaria to the overall morbidity and mortality varies markedly with season. The vast majority of patients received empirical antibiotic therapy during hospital stay, even in the absence of clinical or laboratory evidence of bacterial infection. Better diagnostics are urgently needed to improve clinical management of febrile illnesses and reduce antibiotic overuse.

## Funding

This work was supported by Fundação para a Ciência e a Tecnologia, UID/04413/2020, LA‐REAL LA/P/0117/2020; Conselho Nacional de Desenvolvimento Científico e Tecnológico, 303039/2024‐8: Coordenação de Aperfeiçoamento de Pessoal de Nível Superior (CAPES), Brazil.

## Ethics Statement

The study protocol was approved by the Ethics Review Board of the Central Hospital Dr. Agostinho Neto and the Children's Hospital Pioneiro Zeca, Lubango, Angola. Written informed consent (and assent for children) was obtained at enrollment from patients or their parents/caregivers.

## Conflicts of Interest

The authors declare no conflicts of interest.

## Supporting information


**Table S1:** Final diagnosis assigned by attending clinicians to 849 patients presenting with a febrile illness in Lubango city, Angola, 2023–2024.

**Table S2:** Causes of fever identified at enrollment and day‐28 clinical outcomes, Lubango city, Southern Angola, 2023–2024.

## Data Availability

The de‐identified dataset analyzed in this study is available at the University of São Paulo data repository (https://uspdigital.usp.br/repositorio/). Researchers interested in potential collaboration should contact the corresponding author (muferrei@usp.br).
