# Supplementary material for: Age‐Related and Seasonal Variation in Malaria and Other Causes of Fever and Their Association With Clinical Outcomes in Southern Angola: A Hospital‐Based Study
Source: Trop Med Int Health. 2026 May 16;31(9):1273–83. doi: 10.1111/tmi.70166 (PMC13533634; doi:10.1111/tmi.70166)
Supplement: Supplementary file 1 [file TMI-31-1273-s001.docx]

|  | **Day-28 follow-up** | | | |
| --- | --- | --- | --- | --- |
|  |  | **Complete recovery** | **Partial recovery** | **Worse than day 0 or death** |
| **Cause of fever identified at enrollment** | **No. of patients** | **No. (%)** | **No. (%)** | **No. (%)** |
| Upper respiratory tract infection | 109 | 107 (98.2%) | 2 (1.8%) | 0 (0.0%) |
| Lower respiratory tract infection | 141 | 67 (47.5%) | 51 (36.2%) | 23 (16.3%) |
| Gastrointestinal or hepatic infection | 138 | 94 (68.1%) | 28 (20.3%) | 16 (11.6%) |
| Genitourinary infection | 43 | 34 (79.1%) | 6 (13.9%) | 3 (7.0%) |
| Central nervous system infection | 22 | 9 (40.9%) | 3 (13.6%) | 10 (45.4%) |
| Skin/joint/mucosal infection | 38 | 30 (78.9%) | 5 (13.2%) | 3 (7.9%) |
| Fever with another focus or no focus identified | 102 | 29 (28.4%) | 61 (59.8%) | 12 (11.8%) |
| Positive microscopy for malaria | 273 | 222 (81.3%) | 4 (1.5%) | 47 (17.2%) |
| **Total** | 818 | 558 (68.2%) | 157 (19.2%) | 103 (12.6%) |

**Supplementary Table S2.** Causes of fever identified at enrollment and day-28 clinical outcomes, Lubango city, Southern Angola, 2023-24.

The most frequent day-28 outcome per cause of fever is underlined.
