# Supplementary material for: Age‐Related and Seasonal Variation in Malaria and Other Causes of Fever and Their Association With Clinical Outcomes in Southern Angola: A Hospital‐Based Study
Source: Trop Med Int Health. 2026 May 16;31(9):1273–83. doi: 10.1111/tmi.70166 (PMC13533634; doi:10.1111/tmi.70166)
Supplement: Supplementary file 2 [file TMI-31-1273-s002.docx]

**Supplementary Table 1.** Final diagnosis assigned by attending clinicians to 849 patients presenting with a febrile illness in Lubango city, Angola, 2023-24.

| **Cause of fever identified at enrollment** | **Final diagnosis** | **Number** |
| --- | --- | --- |
| **Lower respiratory tract infection**  **(n = 166)** | Community-acquired pneumonia (CAP) | 86 |
|  | CAP and malaria | 14 |
|  | CAP and HIV infection | 2 |
|  | CAP and acute diarrheal disease (ADD) | 1 |
|  | CAP and typhoid fever | 1 |
|  | CAP and scarlet fever | 1 |
|  | CAP, oropharyngeal candidiasis and HIV infection | 1 |
|  | CAP, malaria, dengue and urinary schistosomiasis | 1 |
|  | Bronchopneumonia | 9 |
|  | Bronchiolitis | 1 |
|  | Acute bronchitis | 3 |
|  | Pulmonary tuberculosis (Tb) | 30 |
|  | Pulmonary Tb and HIV infection | 4 |
|  | Pulmonary Tb and dengue | 1 |
|  | Pulmonary Tb, oropharyngeal candidiasis and HIV infection | 1 |
|  | Pulmonary Tb and CAP | 1 |
|  | Miliary Tb | 2 |
|  | Miliary Tb and HIV infection | 1 |
|  | Miliary Tb, ADD and HIV infection | 1 |
|  | Miliary Tb, malaria and HIV infection | 1 |
|  | Pleural Tb | 2 |
|  | CAP peritoneal Tb and HIV infection | 1 |
|  | *Streptococcus* sepsis and HIV infection | 1 |
| **Upper respiratory tract infection**  **(n = 110)** | Acute nasopharyingitis | 57 |
|  | Acute pharyngotonsillitis | 38 |
|  | Acute pharyngotonsillitis and dengue | 3 |
|  | Acute pharyngotonsillitis and scarlet fever | 2 |
|  | Acute pharyngotonsillitis and malaria | 1 |
|  | Acute pharyngotonsillitis and intestinal amebiasis | 1 |
|  | Acute laryngitis | 4 |
|  | Acute parotitis | 3 |
|  | Common cold and malaria | 1 |
| **Gastrointestinal or hepatic infection**  **(n = 142)** | Acute diarrheal disease (ADD) | 39 |
|  | ADD and HIV infection | 6 |
|  | ADD and malaria | 2 |
|  | CAP and ADD | 1 |
|  | Miliary Tb, ADD and HIV infection | 1 |
|  | Typhoid fever | 34 |
|  | Typhoid fever and malaria | 4 |
|  | Typhoid fever and dengue | 1 |
|  | CAP and typhoid fever | 1 |
|  | Typhoid fever with intestinal perforation | 9 |
|  | Acute gastroenteritis | 18 |
|  | Peritoneal Tb | 6 |
|  | CAP, peritoneal Tb, and HIV infection | 1 |
|  | Bacterial peritonitis | 4 |
|  | Spontaneous bacterial peritonitis, malaria and hepatitis B | 1 |
|  | *Salmonella* sepsis | 2 |
|  | Acute viral hepatitis | 2 |
|  | Acute hepatitis B and malaria | 1 |
|  | Hepatitis C and malaria | 1 |
|  | Acute appendicitis | 1 |
|  | Acute pharyngotonsillitis and intestinal amebiasis | 1 |
|  | Intestinal parasitism and malaria | 2 |
|  | HIV infection | 1 |
|  | Intestinal hernia and bacterial sepsis | 1 |
|  | No specific infection identified (NSII) | 2 |
| **Genitourinary infection**  **(n = 43)** | Urinary tract infecton, no site specified | 15 |
|  | Acute pyelonephritis | 7 |
|  | Acute pyelonephritis and sepsis | 4 |
|  | Acute pyelonephritis and malaria | 3 |
|  | Urinary schistosomiasis | 4 |
|  | Urinary schistosomiasis and CAP | 1 |
|  | Urinary schistosomiasis and malaria | 1 |
|  | Urinary schistosomiasis, dengue, malaria and CAP | 1 |
|  | Pelvic inflammatory disease | 2 |
|  | Cystitis | 1 |
|  | Severe malaria and acute renal failure | 1 |
|  | Acute prostatitis | 1 |
|  | Leptospirosis | 1 |
|  | Genital herpes and HIV infection | 1 |
| **Central nervous system infection**  **(n = 22)** | Bacterial meningitis | 5 |
|  | Bacterial meningitis and HIV infection | 1 |
|  | Bacterial meningitis and neurocysticercosis | 1 |
|  | Bacterial meningitis, malaria and HIV infection | 1 |
|  | Viral meningoencephalytis | 5 |
|  | Viral meningoencephalytis and malaria | 1 |
|  | Viral encephalytis and HIV infection | 1 |
|  | Viral meningitis | 3 |
|  | Viral meningitis and malaria | 1 |
|  | Viral meningitis and dengue | 1 |
|  | Neurocysticercosis | 1 |
|  | Neurotuberculosis and malaria | 1 |
| **Skin/joint/mucosal infection**  **(n = 38)** | Facial cellulitis | 6 |
|  | Facial cellulitis and pharyngeal abscess | 1 |
|  | Facial cellulitis and malaria | 1 |
|  | Facial cellulitis, necrotizing fasciitis and sepsis | 1 |
|  | Cellulitis, another site | 1 |
|  | Acute otitis media | 4 |
|  | Acute otitis media and malaria | 2 |
|  | Perichondritis | 1 |
|  | Necrotizing fasciitis | 1 |
|  | Furuncle | 1 |
|  | Perianal abscess | 1 |
|  | Gluteal abscess and HIV infection | 1 |
|  | Psoas abscess and HIV infection | 1 |
|  | Scarlet fever | 1 |
|  | Acute pharyngotonsillitis and scarlet fever | 2 |
|  | Oropharyngeal candidiasis | 1 |
|  | Oropharyngeal candidiasis and HIV infection | 1 |
|  | CAP, oropharyngeal candidiasis and HIV infection | 1 |
|  | Pulmonary Tb, oropharyngeal candidiasis and HIV infection | 1 |
|  | Viral exanthem, unespecified | 2 |
|  | Roseola | 2 |
|  | Chickenpox | 2 |
|  | Measles | 1 |
|  | Herpes zoster | 1 |
|  | Erythema multiforme due to herpes simplex infection | 1 |
| **Fever with another focus or no focus identified (n = 103)** | No specific infection identified (NSII) | 48 |
|  | NSII, presumptive treatment with antimalarials | 37 |
|  | Fever of unknown origin | 2 |
|  | Infectious mononucleosis | 5 |
|  | Primary HIV infection | 3 |
|  | Hemorrhagic fever of unknown etiology | 1 |
|  | Dengue | 2 |
|  | Chikungunya fever | 1 |
|  | Acute pericarditis, unknown etiology | 1 |
|  | Neurocysticercosis | 1 |
|  | Q fever, presumed | 1 |
|  | Kawasaki disease, presumed | 1 |
| **Positive microscopy for malaria**  **(n = 275)** | Malaria | 228 |
|  | CAP and malaria | 14 |
|  | Malaria and dengue | 3 |
|  | Acute pyelonephritis and malaria | 3 |
|  | Typhoid fever and malaria | 4 |
|  | Acute otitis media and malaria | 2 |
|  | Intestinal parasitism and malaria | 2 |
|  | ADD and malaria | 2 |
|  | Malaria and HIV infection | 2 |
|  | Acute hepatitis B and malaria | 2 |
|  | Hepatitis C and malaria | 1 |
|  | Viral meningoencephalytis and malaria | 1 |
|  | Viral meningitis and malaria | 1 |
|  | Bacterial meningitis, malaria and HIV infection | 1 |
|  | Neurotuberculosis and malaria | 1 |
|  | Malaria, HIV infection and chronic liver disease | 1 |
|  | Miliary Tb, malaria and HIV infection | 1 |
|  | Spontaneous bacterial peritonitis, malaria and hepatitis B | 1 |
|  | Common cold and malaria | 1 |
|  | Malaria and parotitis | 1 |
|  | Facial cellulitis and malaria | 1 |
|  | Urinary schistosomiasis and malaria | 1 |
|  | CAP, malaria, dengue and urinary schistosomiasis | 1 |
